# The Practical Medicine Series

**Published:** 1903-11

**Authors:** 


The Practical Medicine Series.—June, 1903. Part I. Pediatrics. Edited by Isaac A. Abt, M. D., Assistant Professor, Rush Medical College. Part II. Orthopedic Surgery. Edited by John Ridlon, A. M., M. D., of Northwestern University Medical School. August, 1903. Physiology, Pathology, Bacteriology, Anatomy, and a Dictionary of New Words. Price of each volume, $1.25, or $7.50 per set. Published by Year Book Publishing Co., 40 Dearborn Street, Chicago.

The general arrangement of this series has several times been discussed in the Journal. Suffice to say that for brevity, conciseness, breadth of view and thorough up-to-dateness this series of books is almost without parallel in the whole range of medical literature. Every practicing physician should not only possess but read and mentally digest the contents of each one of these little books. J. M. L.

